# Sorption of Polycyclic Aromatic Sulfur Heterocycles (PASH) on Nylon Microplastics at Environmentally Relevant Concentrations

**DOI:** 10.3390/molecules29071653

**Published:** 2024-04-07

**Authors:** Stephanie D. Nauth, Andres D. Campiglia

**Affiliations:** Department of Chemistry, University of Central Florida, Physical Sciences Building. 4111, Orlando, FL 32816, USA; st655863@ucf.edu

**Keywords:** polycyclic aromatic sulfur heterocycles, sorption, sorption uptake, kinetics, nylon microplastics

## Abstract

Microplastics have garnered an infamous reputation as a sorbate for many concerning environmental pollutants and as a delivery vehicle for the aquatic food chain through the ingestion of these contaminated small particulates. While sorption mechanisms have been extensively studied for polycyclic aromatic hydrocarbons, polycyclic aromatic sulfur heterocycles (PASHs) have not been investigated, partly due to their low concentrations in aquatic ecosystems. Herein, an analytical methodology is presented for the analysis of dibenzothiophene, benzo[b]naphtho[1,2-b]thiophene, benzo[b]naphtho[2,1-b]thiophene, benzo[b]naphtho[2,3-b]thiophene, chryseno[4,5-bcd]thiophene and dinaphtho[1,2-b:1′,2′-d]thiophene at relevant environmental concentrations based on solid phase extraction and high-performance liquid chromatography. The sorption uptake behavior and the sorption kinetics of the three benzo[b]napthothiophene isomers were then investigated on nylon microplastics to provide original information on their environmental fate and avoid human contamination through the food chain. The obtained information might also prove relevant to the development of successful remediation approaches for aquatic ecosystems.

## 1. Introduction

Microplastics have been a concern in aquatic environments throughout the years. With dimensions ranging from 0.1 to 5000 μm, the small size of these particulates makes their removal from environmental waters challenging [1] and facilitates their ingestion by aquatic organisms [2]. The rather large surface area of microplastics promotes the uptake of toxic contaminants from aquatic environments and enhances their role as chemical vectors for food chain contamination [3]. For instance, specific surface areas for polyamide microplastics have been reported in the range of 0.017–0.544 m^2^/g for particles sizes varying from 4000 to 29.7 µm, respectively [4]. Organic pollutants known to sorb onto microplastics include polycyclic aromatic hydrocarbons (PAHs) [3], pharmaceuticals [5], polychlorinated biphenyl [6] and polyhalogenated carbazole [7]. Their sorption onto microplastics occurs due to several retention mechanisms, which include a combination of pore filling, hydrogen bonding, pi-pi interactions, electrostatic interactions and van der Waals forces [8].

The presented studies deal with the sorption of polycyclic aromatic sulfur heterocycles (PASHs) onto nylon microplastics. Similar to their parent homocyclic species (PAHs), which contain only carbon and hydrogen, PASHs are usually formed during the incomplete combustion or pyrolysis of organic matter. In addition to their mutagenic and carcinogenic properties, some of them appear on pollutant lists around the world [9,10,11]. Specifically relevant to food chain contamination is the exacerbated ability of PASHs to bioaccumulate in aquatic organisms [12], which results from the presence of the sulfur atom in their heterocyclic molecular structure.

According to our literature search, this is the first report on the sorption of PASHs onto microplastics. To achieve low concentrations of PASHs in aqueous environments [13,14], a method was developed based on solid-phase extraction (SPE) and high-performance liquid chromatography (HPLC) with fluorescence detection. The pre-concentration factors achieved with the SPE procedure, and the appropriate choice of excitation and emission wavelengths made the analysis of several PASHs at parts per trillion (ppt) concentration levels possible. These included dibenzothiophene (DBT), benzo[b]naphtho[1,2-b]thiophene (BbN12T), benzo[b]naphtho[2,1-b]thiophene (BbN21T), benzo[b]naphtho[2,3-b]thiophene (Bb23T) chryseno[4,5-bcd]thiophene (C45T) and dinaphtho[1,2-b:1′,2′-d]thiophene (DiN1212T).

The SPE-HPLC method was then applied to study the sorption uptake and sorption kinetics of BbN12T, BbN23T and BbN21T onto nylon 11 and nylon 6,12. Nylon or polyamide is a popular type of plastic used in fishing gear for both commercial and recreational purposes. Since 18% of marine plastic waste is due to the fishing industry [15], the present studies provide relevant information regarding the contamination issue of microplastics in aquatic ecosystems.

## 2. Results and Discussion

### 2.1. HPLC Analytical Figures of Merit

HPLC conditions were optimized for the separation and detection of the six PASHs studied in this investigation. Figure S1 shows a typical chromatogram obtained from a synthetic mixture with the separation conditions outlined in Section 3.3. Since complete separation was obtained in all cases, these chromatographic conditions were kept constant for the remainder of this study.

The excitation and emission wavelengths for fluorescence detection were selected from signal-to-noise (S/N) ratios measured at the maximum intensities of chromatographic peaks (S) and the baseline noise (N) collected for 0.5 min prior to each chromatographic peak in the chromatograms. Table S1 summarizes the excitation and emission wavelengths that provided the highest S/N ratio for each PASH, along with their time window for chromatographic elution.

The analytical figures of merit for HPLC analysis are summarized in Table 1. No attempts were made to experimentally determine the upper concentration limit of the linear dynamic range. The correlation coefficients that are close to unity confirm the linear dynamic ranges (LDRs) with at least two orders of magnitude and the relative standard deviations (RSDs) demonstrate the good reproducibility of measurements at the parts-per-billion (ng/mL) concentration levels. The limits of detection (LODs) and the limits of quantitation (LOQs) were below 1 and 3 ng/mL, respectively. These LODs and LOQs are well above the parts-per-trillion concentration levels of PASHs in aquatic ecosystems [13,14].

### 2.2. HPLC-SPE Analytical Figures of Merit

Figure S2 provides a schematic diagram of the SPE procedure coupled to HPLC to achieve environmental concentrations of PASHs. The use of heptanol prior to sample pre-concentration removed unknown fluorescence impurities present in the SPE cartridges that co-eluted with several PASHs. 

Figure S3 shows a typical SPE-HPLC chromatogram obtained from the pre-concentrated sample. Table 2 summarizes the SPE-HPLC AFOMs for the studied compounds. As expected, the retentions times were consistent with those in Table 1 and the LODs and LOQ were improved by at least one order of magnitude. The LDRs of the calibration curves and the acceptable reproducibility of measurements (RSD at medium linear concentrations demonstrate the ability to perform an SPE-HPLC analysis of PASHs at environmentally relevant concentration levels). Although BbN12T was the only PASH to present an analytical recovery statistically equivalent to 100% (t_exp_ = 1.98, t_crit_ = 4.30, P = 95%; N = 3), the low standard deviations of all the analytical recoveries show the satisfactory precision of measurements for pursuing sorption studies at environmentally relevant concentrations.

### 2.3. Sorption Uptake by Nylon Microplastics

BbN12T, BbN23T and BbN21T were selected for these studies. These three PASHs have the same molecular weight (234 g·mol^−1^) and the same water solubility (2.291 mmol/L) [16,17,18]. Their molecular lengths (L) and thicknesses (T) varied as follows: BbN12T (L = 12.72 Å and T = 4.39 Å), BbN21T (L = 13.66 Å and T = 4.06 Å) and BbN23T (L = 13.90 Å and T = 4.06 Å) [19,20].

Aqueous solutions containing the three isomers and microplastic particles were thoroughly shaken for various exposure times and the isomer concentrations were monitored via SPE-HPLC. The sorption uptake (q_p_) of each PASH was calculated with the following formula [21]:

(1) $$q_{p}=\frac{\left(c_{t}-c_{aq}\right)\times 0.100\;L}{m_{p}}$$

where q_p_ represents the mass of PASH adsorbed on the surface of the pellet (ng/g), c_t_ is the initial concentration of each PASH in aqueous solution (ng/L), c_aq_ is the PASH concentration in the aqueous solution after a certain exposure time (ng/L) and m_p_ is the mass of the microplastics (g) used for the experiments.

**Figure 1:**
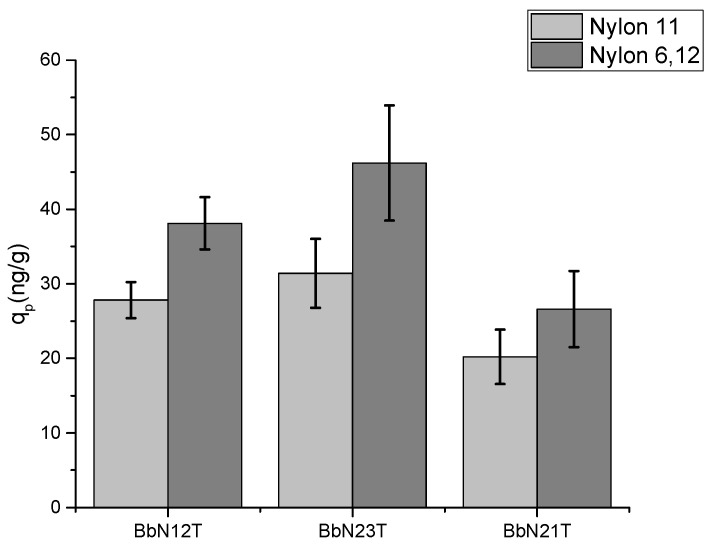
Maximum sorption uptake for BbN12T, BbN23T and BbN21T onto Nylon 11 and Nylon 6,12.

The maximum sorption uptake for the tested plastics is shown in Figure 1. The maximum sorption uptake was determined using Equation (1) and the standard deviations were calculated using the values from Table S2 and the Equation (S1) through Equation (S3). The figure shows that Nylon 6,12 shows a higher mass sorbed for all the PASH isomers. BbN23T showed the highest sorption between both plastics while BbN21T showed the least sorption. These trends indicate that the type of nylon impacts the sorption uptake of these pollutants, and that the pollutant structure also dictates the sorption onto microplastics. In addition, Table S3 shows the masses for the maximum sorption uptake. The masses sorbed for BbN23T and BbN12T are statistically different from each other, while the masses sorbed for BbN21T for both Nylon 11 and Nylon 6,12 are statistically equivalent. 

**Figure 2:**
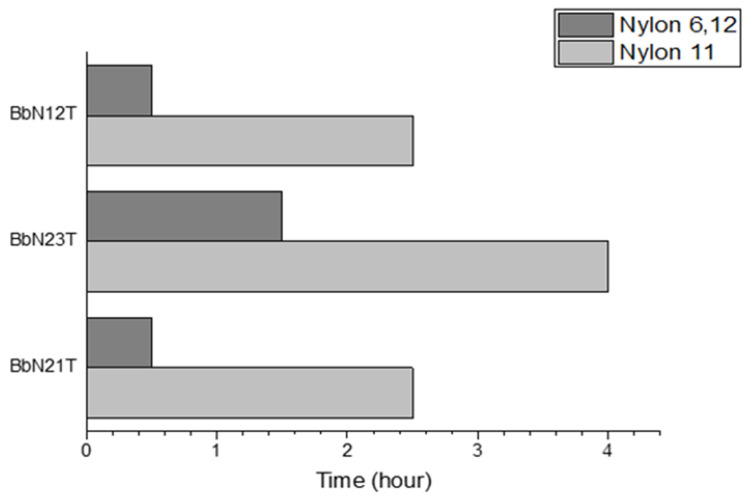
Time taken for maximum sorption to occur of BbN12T, BbN23T and BbN21T onto Nylon 11 and Nylon 6,12.

Figure 2 shows the time needed to achieve the maximum sorption. For all PASHs, Nylon 6,12 was shown to be faster than Nylon 11. With Nylon 6,12, the sorption the maximum sorption occurred in less than 2 h for all three isomers. It took almost more than double the time for Nylon 11 to reach the maximum sorption uptake compared to Nylon 6,12.

### 2.4. Sorption Kinetic Modeling

The sorption of the PASHs onto the microplastics was further analyzed using pseudo-first-order and pseudo-second-order fittings. Both fittings are commonly used to understand the mechanisms driving the sorption. The sorption uptakes established an equilibrium that was further proven by the statistical equivalence of the last two time points.

**Figure 3:**
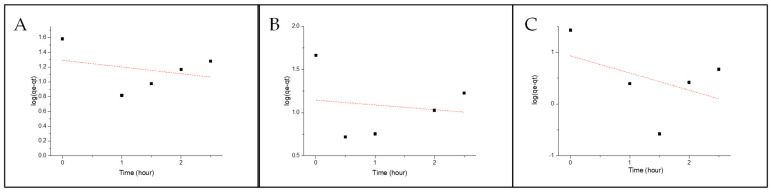
Pseudo-first-order fitting onto Nylon 6,12 for (**A**) BbN12T, (**B**) BbN23T and (**C**) BbN21T.

**Figure 4:**
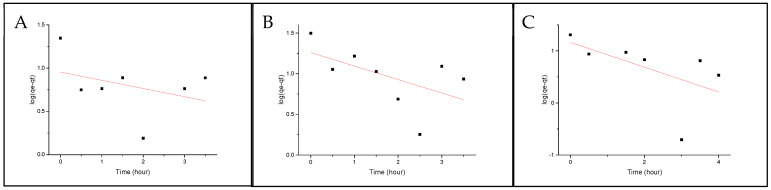
Pseudo-first-order fitting onto Nylon 11 for (**A**) BbN12T, (**B**) BbN23T and (**C**) BbN21T.

When the sorption behavior follows pseudo-first-order, adsorption occurs through diffusion [21]. Pseudo-first-order is represented by Equation (2).

(2) $$\log\left(q_{e}-q_{p}\right)=\log q_{eq,1}-\frac{k_{1}}{2.303}t$$

where q_e_ = mass on the pellet at equilibria (ng/g), q_p_ = mass on pellet after exposure time (ng/g), q_eq,1_ =mass on the pellet at equilibria, determined by the pseudo-first-order model, and k_1_ = rate constant for the pseudo-first-order model. Figure 3 and Figure 4 show the fittings for both Nylon 11 and Nylon 6,12. None of the isomers show any linearity with the model; therefore, it can be concluded that the absorption mechanism is not diffusion-based.

**Figure 5:**
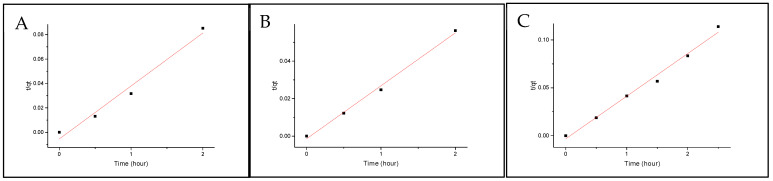
Pseudo Second Order fitting onto Nylon 6,12 for (**A**) BbN12T, (**B**) BbN23T, and (**C**) BbN21T.

**Figure 6:**
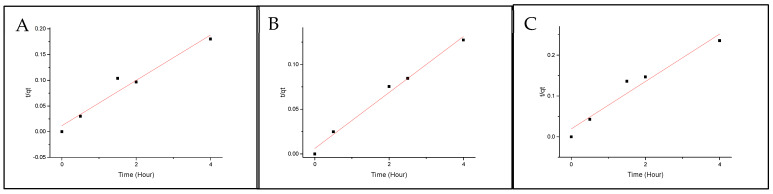
Pseudo-second-order fitting onto Nylon 11 for (**A**) BbN12T, (**B**) BbN23T and (**C**) BbN21T.

Pseudo-second-order kinetic modeling shows that the absorption rate is dependent on the capacity for sorption and not the concentration of the absorbate [21]. This model is represented by the following equation:

(3) $$\frac{t}{q_{t}}=\frac{1}{k_{2}q_{e,model}^{2}}+\frac{1}{q_{e,model}}t$$

where q_e, model_ is the sorption capacity at equilibrium, q_t_ is the amount of the analyte sorbed at each time point, k_2_ is the pseudo-second-order rate constant and k_2_q_e_ is the initial sorption rate. Figure 5 and Figure 6 show the fitting for both Nylon 6,12 and Nylon 11. All the isomers show great linearity, which indicates good agreement with the model.

Table 3 summarizes the rate constants and the initial sorption rates. Nylon 6,12 showed a higher initial sorption rate than Nylon 11. The model predicts that BbN23T will have the highest sorption capacity at equilibrium, which is confirmed by the sorption uptake showing that BbN23T has the largest uptake on the microplastics.

## 3. Materials and Methods

### 3.1. Materials and Chemicals

DBT and BbN12T (purity = 99%) were purchased from Sigma Aldrich (Milwaukee, WI, USA). BbN23T (purity > 99%) and BbN12T (purity > 99%) were acquired from the European Commission (Brussels, Belgium). C45T and DiN1212T were both obtained from the National Institute Standards and Technology (NIST) (Gaithersburg, MD, USA) and used as-received. The molecular structures for these compounds are presented in Figure S4.

Nylon 11 and Nylon 6–12 were obtained from Sigma Aldrich as pellets, with particle sizes of 3 mm and 2 mm, respectively. The molecular structures for these compounds are shown in Supplemantal Information Figure S5. Physicochemical properties for both nylons are displayed in Table S4 [22,23,24]. The averaged dimensions of the microplastic pellets used are shown in Table S5.

HPLC-grade n-hexane, n-heptane, n-octane, n-nonane and methanol were acquired from Sigma Aldrich. HPLC-grade heptanol was purchased from Acros Organic (Geel, Belgium). HPLC-grade acetonitrile (ACN) was obtained from VWR (Radnor, PA, USA). Nanopure water obtained from a Barnstead Nanopure Infinity (Dubuque, IA, USA) water system was used throughout all the experiments. Waters (Milford, MA, USA) Sep-Pak Plus C-18 cartridges were used on a vacuum manifold.

### 3.2. Stock Preparation

Stock solutions of PASHs were prepared with the following solvents: n-heptane (DBT) n-octane (BbN12T, BbN21T, C45T and DiN1212T) and n-nonane (BbN23T). All stock solutions were kept in the dark at 4 °C. Working solutions of pure PASHs were prepared daily via evaporation of the n-alkane solvent under a gentle stream of nitrogen gas (100% purity). In all cases, the reconstituting solvent was ACN.

### 3.3. HPLC Analysis

All PASHs concentrations were monitored via high-performance liquid chromatography (HPLC) with an HPLC system from Hitachi (San Jose, CA, USA). Its main components were an L-7100 mobile phase pump, an L-7485 fluorescence detector and an L-761 on-line degasser. The entire system was computer-controlled with D-7000 HPLC Software Manager. All separations were made using a Zorbax Eclipse PAH column with a 4.6 mm length, 250 mm diameter and 5 μm average particle diameters. All sample injections were held constant at 20 μL using a fixed-volume injection loop. Laboratory reagent blanks were run with each series of samples under identical experimental conditions. Supplemantal Information Table S1 shows the wavelengths used during the HPLC analysis.

### 3.4. Solution Preparation for HPLC Analytical Figures of Merit

Calibration curves were constructed with synthetic mixtures containing DBT, BbN12T, BbN21T, C45T, BbN23T and DiN1212T at various concentrations. All the mixtures were made in 100% ACN to match the mobile phase selected for HPLC. This was accomplished by evaporation of the n-alkane PASH solvent under a gentle stream of nitrogen gas and reconstitution with ACN.

### 3.5. Preparation for SPE-HPLC Analytical Figure of Merit

Synthetic mixtures containing DBT, BbN12T, BbN21T, C45T, BbN23T and DiN1212T were prepared for the experiments with PASHs concentrations varying from 25 to 100 parts per trillion. The solvent composition of all the mixtures was 99.5% H_2_O/0.5% ACN. This was accomplished by spiking 100 mL of H_2_O with microliter volumes of PASHs solutions in ACN. SPE followed the procedure outlined in Supplemantal Information Figure S3.

### 3.6. Sorption Experiments

Synthetic mixtures for these experiments contained BbN12T, BbN23T and BbN21T, with final concentrations at the 50 pg/mL level. Their solvent composition was 99.5% H_2_O/0.5% ACN. This was accomplished by spiking 100 mL of H_2_O with microliter volumes of PASHs solutions in ACN. Six (6) pellets of each plastic consisting of approximately 150 mg of Nylon 11 and 100 mg of Nylon 6,12 were added to 100 mL of the aqueous solution and shaken for various exposure times, which ranged from 0.5 h to 4 h. An SPE-HPLC analysis of the aqueous solution was conducted at 0.5, 1, 1.5, 2, 2.5, 3, 3.5 and 4 h. An SPE-HPLC analysis of synthetic mixtures shaken in the absence of microplastics was run in parallel at all time intervals.

## 4. Conclusions

The SPE-HPLC method developed in these studies makes it possible to determine PASH in aqueous solutions at pg/mL concentration levels. To reach such low concentration levels, excitation and fluorescence wavelengths were optimized for each of the studied fluorophores. By doing so, excellent analytical figures of merit were obtained at relevant environmental concentrations. With the new method, the sorption uptake of benzonaphthothiophene isomers onto nylon microplastics was demonstrated for the first time. In all cases, the nylon type and the isomer structure played a role in the sorption uptake, which followed pseudo-second-order kinetics. Our findings, therefore, indicate that the interaction of PASH with microplastics in aquatic environments deserves more attention from both the remediation and the toxicological perspectives.

## Figures and Tables

**Table 1:** HPLC analytical figures of merit.

PASH	Retention Time ^1^ (min)	LOD ^2^ (ng/mL)	LOQ ^3^ (ng/mL)	LDR ^4^ (ng/mL)	% RSD ^5^
DBT	3.87 ± 0.022	0.9	3.0	3.0–200	5.64
BbN12T	5.68 ± 0.078	0.3	0.9	0.9–200	3.53
BbN23T	7.07 ± 0.135	0.2	0.8	0.8–200	0.30
BbN21T	8.78 ± 0.196	0.3	0.9	0.9–200	1.94
C45T	13.24 ± 0.346	0.5	1.8	1.8–200	4.10
DiN1212T	26.33 ± 0.868	0.7	2.2	2.2–200	3.75

^1^ Retention times are the average of three sample injections; sample volume = 20 µL. ^2^ Limit of detection (LOD) was calculated using LOD = 3S_B_/m, where S_B_ is the standard deviation of the blank using the following formula: 1/5 (*Noise*_*max*_ − *Noise*_*min*_). The noise was recorded for 0.5 min at the base peak of each chromatographic peak. ^3^ Limit of quantitation (LOQ) was calculated using LOQ = 10S_B_/m. ^4^ Linear Dynamic Range (LDR), in ng/mL, extends from the LOQ to an arbitrarily chosen upper linear concentration. ^5^ Relative Standard Deviation (RSD) = S/I × 100, where I is the average intensity and S is the standard deviation of the intensity calculated from three measurements at the middle concentration.

**Table 2:** SPE−HPLC analytical figures of merit.

PASH	Retention Time ^1^ (min)	LOD ^2^ (pg/mL)	LOQ ^3^ (pg/mL)	LDR ^4^ (pg/mL)	RSD ^5^ (%)	Analytical Recovery ^6^ (%)
DBT	3.73± 0.015	0.05	0.2	0.2–75	5.0	90.5 ± 3.1
BbN12T	5.57 ± 0.120	3	10	10–75	1.1	101.6 ± 1.4
BbN23T	7.02 ± 0.125	0.7	2	2–100	3.0	88.2 ± 3.4
BbN21T	8.72 ± 0.159	0.6	2	2–100	3.9	77.0 ± 2.7
C45T	13.11 ±0.405	2	8	8–75	7.4	68.5 ± 4.1
DiN1212T	25.89 ± 0.674	2	7	7–75	1.1	69.9 ± 2.5

^1^ Retention Times are the average of three sample injections; sample volume = 20 µL. ^2^ Limit of detection (LOD) was calculated using LOD = 3S_B_/m, where S_B_ is the standard deviation of the blank using the following formula: $\frac{1}{5}\left(Noise_{max}-{Noise}_{min}\right).$ The noise was recorded for 0.5 min at the base peak of each chromatographic peak. ^3^ Limit of quantitation (LOQ) was calculated using LOQ = 10S_B_/m. ^4^ Linear Dynamic Range (LDR) in ng/mL; extends from the LOQ to an arbitrarily chosen upper linear concentration. ^5^ Relative Standard Deviation (RSD) = S/I × 100, where I is the average intensity and S is the standard deviation of the intensity calculated from three measurements at the middle concentration. ^6^ Analytical recoveries (AR) were calculated with the formula AR = C_S_/C_M_ × 100, where Cs is a medium linear concentration of the standard submitted to the entire experimental procedure and C_M_ is the concentration obtained with the method. The reported values are the averages of three experimental runs.

**Table 3:** Pseudo-second-order fitting parameters.

Type of Microplastics	PASHs	R^2 a^	$q_{e,model}$ ^b^ (ng/g)	$k_{2}q_{e,model}^{2}$ ^c^ (ng/g) × h	$k_{2}$ ^d^ (g/ng) × h
Nylon 6,12	BbN12T	0.9778	23.87	181.8	0.3191
	BbN23T	0.9953	35.46	714.3	0.5681
	BbN21T	0.9949	24.39	1111	1.8676
Nylon 11	BbN12T	0.9975	22.52	217.4	0.4287
	BbN23T	0.9895	31.95	163.9	0.1606
	BbN21T	0.9515	17.21	51.81	0.1749

^a^ R^2^ = Correlation coefficient obtained by fitting data to the pseudo-second-order model. ^b^ q_e,model_ = PASHs-sorbed amount at equilibrium obtained from the pseudo-second-order model fitting using Equation (2). ^c^ $k_{2}q_{e,model}^{2}\;$ *=* initial sorption rate, obtained from the intercept of Equation (2). ^d^ k_2_ = Rate constant of the pseudo-second-order model, obtained from the intercept of pseudo second-order model’s Equation (2).

## Data Availability

Data is contained within this article and Supplementary Materials.

## Supplementary Materials

The following supporting information can be downloaded at: https://www.mdpi.com/article/10.3390/molecules29071653/s1, Figure S1: HPLC-FL Chromatogram of the PASH. All PASH concentration is 50 ng/mL. Table S1: Summary of Fluorescence wavelengths used for HPLC Detection; Figure S2: Summary of SPE method used in this study; Figure S3: HPLC-FL Chromatogram following SPE extraction procedure, where the concentration of all PASH were 25 pg/mL prior to extraction.; Table S2: Values used for the calculation of the maximum sorption uptake with corresponding standard deviations; Equation (S1)–(S3) Error Propagation; Table S3: Maximum Sorption Uptake Masses and Statistical Comparison, Figure S4: Structures of PASH used in this study; Figure S5: Structures of Nylon 11 and Nylon 6,12 used in this study; Table S4: Physicochemical Properties for Nylon 11 and Nylon 6,12; Table S5: Dimensions of 10 Randomly Selected Nylon Pellets.
