# Supplementary material for: Sorption of Polycyclic Aromatic Sulfur Heterocycles (PASH) on Nylon Microplastics at Environmentally Relevant Concentrations
Source: Molecules. 2024 Apr 7;29(7):1653. doi: 10.3390/molecules29071653 (PMC11013277; doi:10.3390/molecules29071653)
Supplement: Supplementary file 1 [file molecules-29-01653-s001.zip › molecules-2920982-supplementary.pdf]

# Supplemental Information

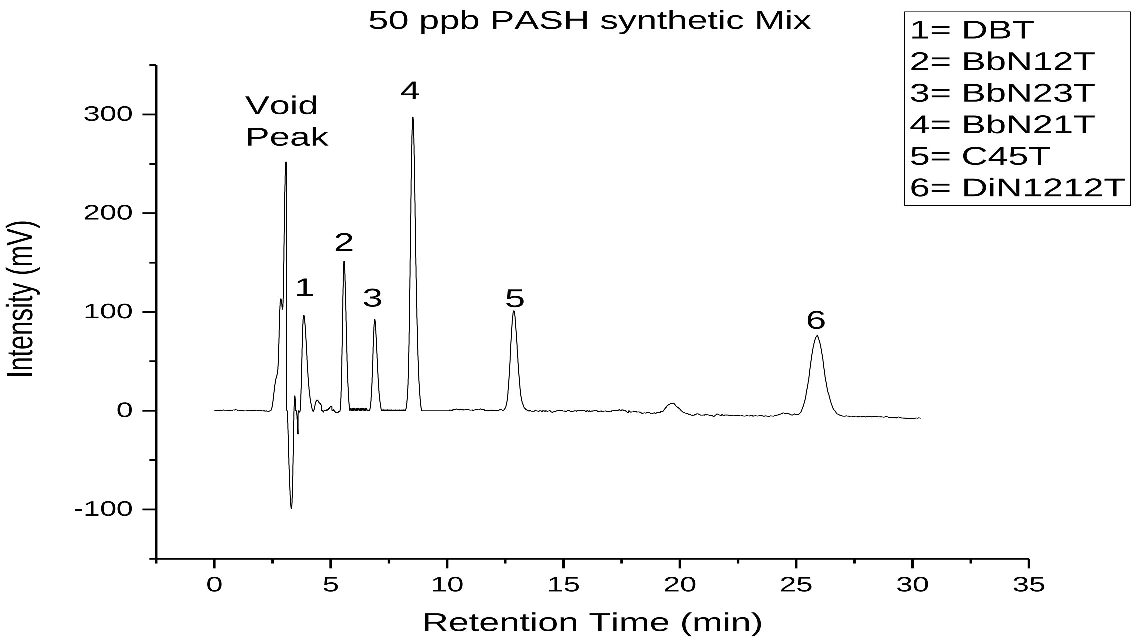


Figure S1: HPLC-FL Chromatogram of the PASH. All PASH concentration is 50 ng/mL.

Table S1: Summary of Fluorescence wavelengths used for HPLC Detection

| PASH | Excitation/ Emission Wavelength (nm) | Time Range (min) |
|---|---|---|
| DBT | 286/342 | 0-4.7 |
| BbN12T | 264/352 | 4.8-6.4 |
| BbN23T | 274/376 | 6.5-8.0 |
| BbN21T | 277/366 | 8.1-10.0 |
| C45T | 359/385 | 10.1-16.1 |
| DiN1212T | 275/381 | 16.1-25.0 |

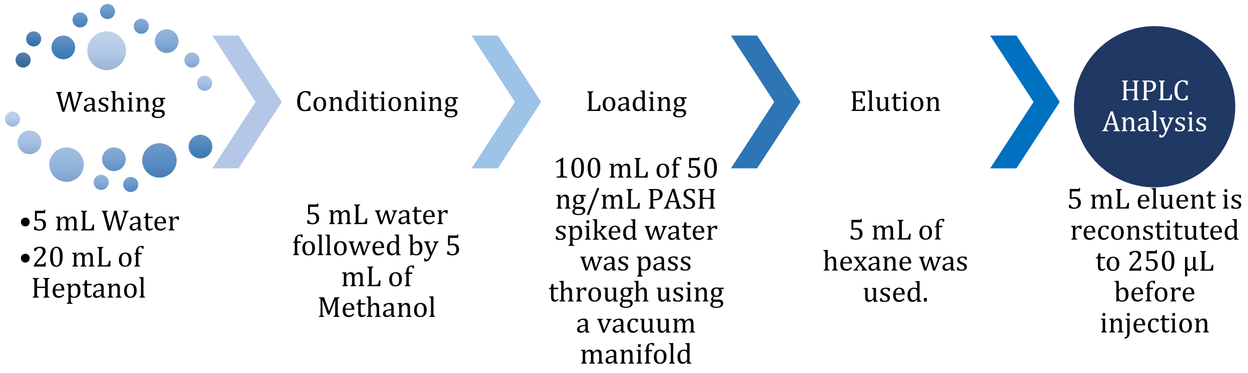


Figure S2: Summary of SPE method used in this study. The flow rate used was 1-2 mL/min.

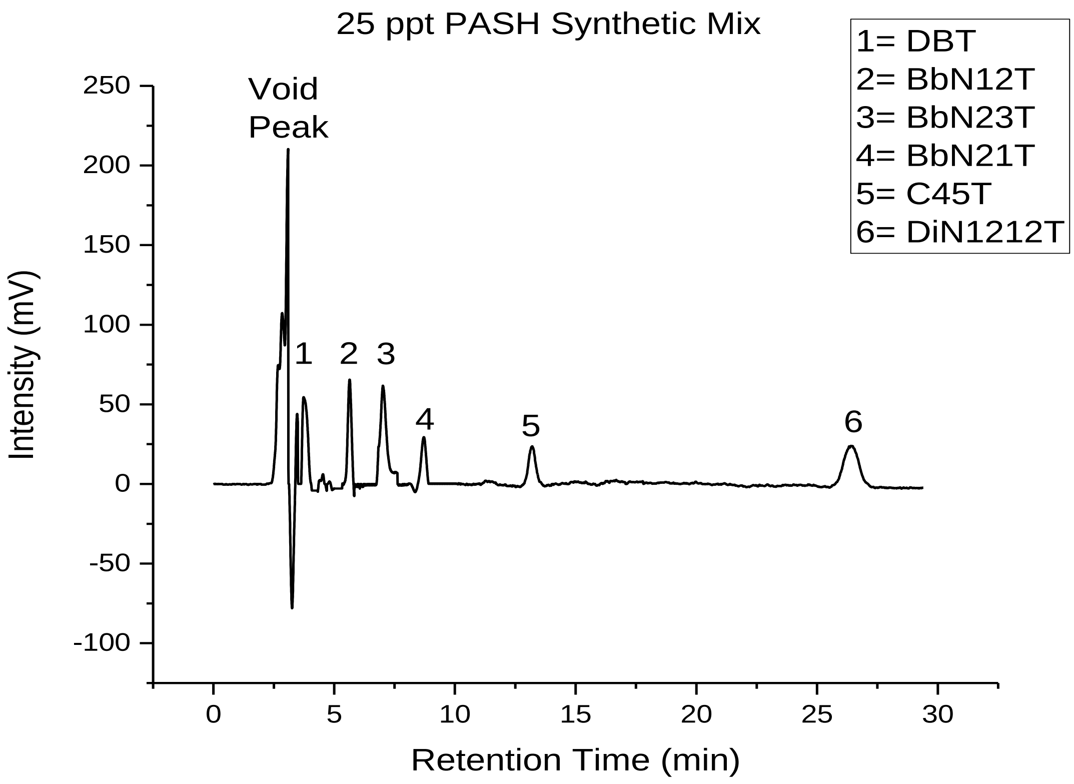


Figure S3: HPLC-FL Chromatogram following SPE extraction procedure, where the concentration of all PASH were 25 pg/mL prior to extraction.

Table S2: Values used for the calculation of the maximum sorption uptake with corresponding standard deviations.

| **Nylon Type** | **PASH** | **Time (hr)** | **$C_T$ (ng/L)** | **$s_{CT}$** | **$C_{aq}$ (ng/L)** | **$s_{Caq}$** | **$C_T$-$C_{aq}$ (ng/L)** | **$s_{(CT-Caq)}$** | **$q_p$ (ng/g)** | **$s_{qp}$** |
|---|---|---|---|---|---|---|---|---|---|---|
| Nylon 11 | BbN12T | 2.5 | 81.99 | 2.91 | 40.11 | 2.19 | 41.88 | 3.64 | 27.82 | 2.43 |
| | BbN23T | 4.0 | 99.51 | 5.69 | 48.05 | 4.75 | 51.46 | 7.40 | 31.41 | 4.61 |
| | BbN21T | 2.5 | 64.64 | 4.11 | 33.94 | 3.62 | 30.70 | 5.48 | 20.20 | 3.64 |
| Nylon 6,12 | BbN12T | 0.5 | 79.80 | 2.84 | 39.40 | 2.19 | 40.40 | 3.59 | 38.11 | 3.52 |
| | BbN23T | 1.5 | 88.11 | 4.81 | 46.38 | 4.79 | 41.73 | 6.97 | 46.19 | 7.72 |
| | BbN21T | 0.5 | 56.51 | 3.63 | 34.72 | 3.75 | 21.79 | 5.22 | 26.61 | 5.12 |

Equations used for error Propagation used in this study:

Equation S1: Error of measurement from the calibration curve (for both $c_t$ and $c_{aq}$)

$$s_c = \frac{S_{y/x}}{b}\sqrt{\frac{1}{m}+\frac{1}{n}+\frac{(y_o-\bar{y})^2}{b^2\sum_i(x_i-\bar{x})^2}}$$

Equation S2: Linear propagation ($q_t$-$q_{aq}$)

$$s_{(c_t-c_{aq})} = \sqrt{s_{c_t}^2 + s_{c_{aq}}^2}$$

Equation S3: Scaling the standard deviation

$$S_{q_p} = \frac{\left(s_{(c_t-c_{aq})}\right)x\,.\,100\;L}{m_p}$$

Legend

Sc= The standard deviation of the concentration

Sy/x= standard deviation of the residual

b= slope

m= number of measurements

n= number of calibration curve points

$y_o - \bar{y}$ = error of the average intensity

$x_i - \bar{x}$= error from the centroid point of the calibration curve

$s_{(c_t-c_{aq})}$= standard deviation of the difference between the total concentration and the aqueous concentration

$s_{c_t}^2$= standard deviation of the total concentration squared

$s_{c_{aq}}^2$=standard deviation of the aqueous concentration squared

$S_{q_p}$= standard deviation of the mass on the pellet per gram of pellet

$m_p$= mass of pellets

Table S3: Maximum Sorption Uptake Masses and Statistical Comparison.

| **PASH** | **Nylon 11 (ng/g)** | **Nylon 6,12 (ng/g)** | **Statistically Equivalent[1]** |
|---|---|---|---|
| BbN12T | 27.82 ± 2.43 | 38.11 ± 3.52 | No ($T_{exp}$= 4.17) |
| BbN23T | 31.41 ± 4.61 | 46.19 ± 7.72 | No ($T_{exp}$= 2.85) |
| BbN21T | 20.20 ± 3.64 | 26.61 ± 5.12 | Yes ($T_{exp}$= 1.70) |

1. T crit= 2.276 (P = 95%; N = 3)

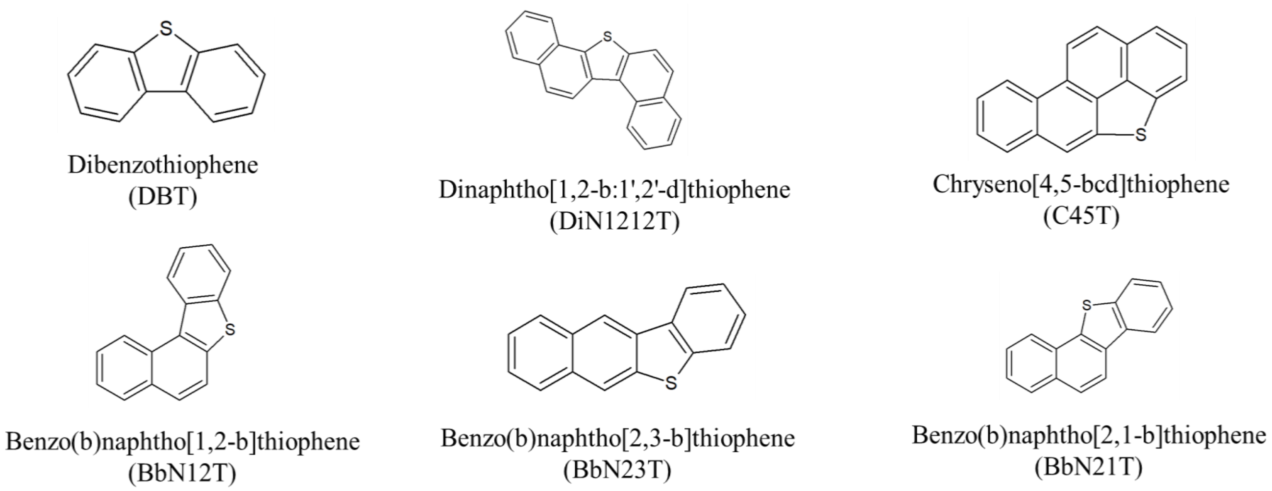


Figure S4: Structures of PASH used in this study

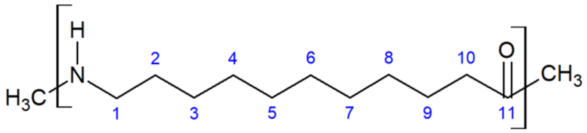


Nylon 11

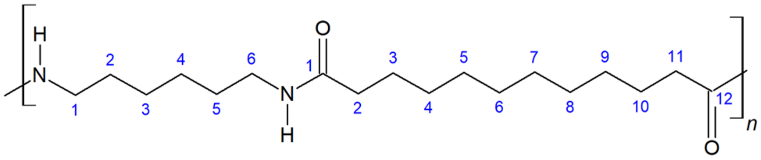


Nylon 6,12

Figure S5: Structures of Nylon 11 and Nylon 6,12 used in this study.

Table S4: Physicochemical Properties for Nylon 11 and Nylon 6,12

| **Physicochemical Properties** | **Nylon 11** | **Nylon 6,12** |
|---|---|---|
| Particle Size | 3 mm[1] | 2 mm[2] |
| Density (at 25 °C) | 1.026 g/mL[1] | 1.300 g/mL[2] |
| Melting Temperature | 198 °C[1] | 218 °C[2] |
| Glass Transition Temperature | 46 °C[2] | 46 °C[2] |
| Length of Monomer | 12.24 Å[3] | 8.83 Å[3,4] /13.31 Å[3,5] |

1. Reference 22
2. Reference 23
3. Reference 24
4. Represents the length between the diamines section.
5. Represents the length between the dicarboxylic section.

Table S5: Dimensions of 10 Randomly Selected Nylon Pellets

| Nylon Type | Length (mm) | Diameter (mm) | Width (mm) | Mass (g) |
|---|---|---|---|---|
| Nylon 11 | 4.4 ± 0.48 | 2.7 ± 0.46 | 3.8 ± 0.40 | 0.027 ± 0.002 |
| Nylon 6,12 | 2.8 ± 0.40 | 2.0 ± 0.00 | 3.2 ± 0.40 | 0.016 ± 0.001 |
